# A phase III double-blind placebo-controlled randomized study of dexamphetamine sulfate for fatigue in primary brain tumors patients: An ANOCEF trial (DXA)

**DOI:** 10.1093/noajnl/vdz043

**Published:** 2019-11-10

**Authors:** Florence Laigle-Donadey, François Ducray, Matthieu Boone, Mamadou Hassimiou Diallo, David Hajage, Carole Ramirez, Olivier Chinot, Damien Ricard, Jean-Yves Delattre

**Affiliations:** 1 AP-HP, Hôpitaux Universitaires La Pitié-Salpêtrière-Charles Foix, Service de Neurologie 2-Mazarin, Paris, France; 2 Hospices Civils de Lyon, Groupe Hospitalier Est, Service de Neuro-Oncologie, Lyon, Cedex, France; 3 Université Claude Bernard Lyon 1; Department of Cancer Cell Plasticity, Cancer Research Centre of Lyon, INSERM U1052, CNRS UMR5286, Lyon, France; 4 Service d’Oncologie Médicale, CHU Amiens-Picardie, Chimère, France; 5 Sorbonne Université, INSERM, Institut Pierre Louis d’Epidémiologie et de Santé Publique, AP-HP, Hôpitaux Universitaires Pitié Salpêtrière Charles Foix, Unité de Recherche Clinique, Paris, France; 6 Sorbonne Université, INSERM, Institut Pierre Louis d’Epidémiologie et de Santé Publique, AP-HP, Hôpitaux Universitaires Pitié Salpêtrière-Charles Foix, Département Biostatistique Santé Publique et Information Médicale, Centre de Pharmacoépidémiologie (Cephepi), Paris, France; 7 Service de Neurochirurgie, Hôpital Roger Salengro, CHRU de Lille, rue Emile Laine, Cedex, France; 8 Aix-Marseille Univ, APHM, CNRS, INP, Inst Neurophysiopathol, CHU Timone, Service de Neuro-Oncologie, Marseille, France; 9 Hôpital d’Instruction des Armées Percy, Service de Santé des Armées, Clamart, France; 10 UMR 8257 MD4 Cognac-G, CNRS, Université Paris Descartes, Service de Santé des Armées, Paris; Ecole du Val-de-Grâce, Service de Santé des Armées, Paris; Centre OncoNeuroTox, Clamart et Paris, France; 11 AP-HP, Hôpitaux Universitaires La Pitié-Salpêtrière-Charles Foix, Service de Neurologie 2-Mazarin; Sorbonne Université, UPMC Univ Paris 06, UMR S 1127; INSERM, U 1127; CNRS, UMR 7225; ICM, Paris, France

**Keywords:** dexamphetamine, fatigue, primary brain tumors

## Abstract

**Background:**

Most patients suffering from a primary brain tumor (PBT) complain of chronic fatigue affecting their quality of life (QOL). We hypothesized that dexamphetamine sulfate, a psychostimulant drug, could improve fatigue in PBT patients.

**Methods:**

A double-blind, phase III, multi-institutional, placebo-controlled randomized trial (1:1 allocation) assessed the efficacy and tolerability of dexamphetamine at a dosage of 30 mg/day in PBT patients with stable disease who complained of severe fatigue, defined as a Multidimensional Fatigue Inventory (MFI-20) score ≥60. The primary outcome was the variation of the MFI 20 score between inclusion and the evaluation at 3 months in nonprogressive patients. Mood, QOL and cognitive function were also evaluated.

**Results:**

From April 2013 to November 2016, 46 patients were enrolled in the study, 41 of whom were evaluable for analysis (dexamphetamine group: 22; placebo group: 19). Tolerance was generally good, with no treatment-related deaths and no grade 4 toxicity. Patients in the dexamphetamine arm complained more frequently of psychiatric side effects (mostly hyperactivity, anxiety, sleep disorder, and irritability) than patients in the placebo arm (*P* = .018). There were no statistically significant differences at 3 months between the dexamphetamine and placebo arms in any of the outcomes (MFI-20, Norris Visual Analog Scale, Hospital Anxiety and Depression Scale (HADS), QOL (EORTC QLQ-C30/BN 20), Marin’s Apathy Evaluation Scale, and cognitive evaluations).

**Conclusion:**

Dexamphetamine at a dosage of up to 30 mg/day for 3 months has acceptable tolerability in PBT patients but does not improve fatigue, cognitive function, or QOL.

**Key Points**

1. The current trial represents the first randomized trial evaluating dexamphetamine in primary brain tumor patients suffering from fatigue.
2. For this reason, despite its negative results, we think that this study could be of interest to the oncological community.

**Importance of the Study**

Fatigue is a key issue in primary brain tumor (PBT) patients. In fact, improving survival is no longer considered sufficient, and research on quality of life, especially fatigue, is continually advancing. To date, as reported in a recent Cochrane review, no treatment has shown efficacy against fatigue in patients suffering from high-grade gliomas.

The current trial represents the first randomized trial evaluating dexamphetamine in PBT patients suffering from fatigue. No significant effect was found. Because of the disappointing influence of psychostimulating agents, other strategies need to be explored to improve fatigue in this population.

The quality of life (QOL) of patients suffering from a primary brain tumor (PBT) is often impaired by severe fatigue caused not only by the cancer but also by the side effects of the treatment.^1^ Fatigue can occur at all stages of the disease, even in the event of response or stabilization of the tumor, and it has debilitating consequences in daily life. Its prevalence in PBT patients is estimated to be between 42% and 90%.^2,3^ Fatigue, cognitive impairment and depression may overlap, and all contribute to affect QOL.^2^

To date, no treatment, including methylphenidate, modafinil, armodafinil, donepezil, or non-pharmacological intervention has shown clear-cut efficacy against fatigue in patients suffering from a PBT. A recent Cochrane review described the current state of this area of research.^2^

Dexamphetamine was found to have promising effects in patients with noncancer-related fatigue syndrome.^4,5^ These early results prompted us to assess the efficacy of dexamphetamine in reducing fatigue in PBT patients with responsive or stabilized tumors in a randomized phase III trial.

## Methods

This double-blind, placebo-controlled randomized trial was approved by the appropriate French legal authority and Ethics Board (Comité de Protection des Personnes d’Ile de France VI) and was conducted in accordance with the ethical standards of the Declaration of Helsinki. The patients signed an informed consent before participating.

### Participants/Eligibility and Exclusion Criteria

Patients who were at least 18 years old and had a Karnofsky Performance Status (KPS) score ≥60% were eligible to participate in the study if they fulfilled the following criteria: (a) Histologically proven PBTs (including gliomas, medulloblastomas, and primary central nervous system [CNS] lymphomas), previously treated with radiotherapy or chemotherapy or both, and a stable or responsive tumor according to RANO criteria. In the case of previous RT, a minimum delay of 3 months after RT was required to exclude pseudoprogression with spontaneous improvement of fatigue. Concurrent chemotherapy was allowed, as well as a stable dose of corticosteroids. (b) Severe fatigue, defined as a Multidimensional Fatigue Inventory (MFI-20) score ≥ 60/100 without concomitant suspected depression (Hospital Anxiety and Depression Scale [HADS] score <8) at inclusion. The exclusion criteria included treatable situations potentially responsible for fatigue (eg, serum hemoglobin < 10 g/dL), severe aphasia or other symptoms making the tests difficult to perform (eg, severe cognitive impairment and right-hand paresis). The exclusion criteria also included all usual contraindications for amphetamines, especially pre-existing personal or familial psychiatric disease, pre-existing cardiovascular disease (or abnormal electrocardiography/echocardiography), glaucoma, hyperthyroidism, drug abuse, hereditary galactose or saccharose intolerance, pregnancy, and Gilles de la Tourette syndrome.

### Trial Design and Treatment

This multi-institutional study was a placebo-controlled randomized trial with a 1:1 allocation. After randomization, each participant received a blister pack of pills containing either the study drug (dexamphetamine 5 mg tablets) or a placebo (identical-appearing pills). Both the study drug and the placebo were taken at the same time, twice daily (5 mg in the morning, 5 mg at noon) for 10 days.

Then, the dose was increased in the event of good tolerance (assessed by a clinical evaluation after 10 days) to the second dosage level (20 mg/day of dexamphetamine or placebo, consisting of 2*5 mg in the morning and 2*5 mg at noon) for another 10 days.

In the event that good tolerance was confirmed (by another clinical evaluation after 3 weeks), participants received the highest dose in the study (30 mg of dexamphetamine or placebo, consisting of 3*5 mg in the morning and 3*5 mg at noon) for an additional 10 days.

Another clinical evaluation of tolerance was performed 1 week later (“Month 1, M1”), and if tolerance was good, the full dose was maintained for the remainder of the study.

Evaluations were performed at baseline, every 10 days during the first month for dose adjustment purposes, and then monthly (M2, M3). Toxic effects were graded using the Common Terminology Criteria for Adverse Effects (CTCAE) version 4.0 and were recorded on a flow sheet by the patients at home between visits.

The procedures for dosage modifications allowed for toxicity. If participants did not tolerate the study agent, the agent was maintained at a lower dosage level or discontinued.

Three months after the beginning of the treatment (M3), patients were clinically evaluated, and their dosage was progressively reduced, with discontinuation of the drug or placebo within 7 days. The patients were then followed up through phone calls to ensure that they had no dependency symptoms.

Regarding the efficacy criteria assessment, detailed below, fatigue, QOL, anxiety, depression (self-administered questionnaires), and cognitive function (cognitive tests performed by a neuropsychologist) were assessed at baseline and 3 months after the beginning of the study. Additionally, at 1 and 2 months after beginning the study, the participants completed self-administered questionnaires regarding anxiety, depression, and QOL.

### Study Outcomes

The primary outcome was the variation of the MFI 20 score between inclusion and the evaluation at 3 months in the case of non-progressive disease during this period.

The secondary outcomes were the occurrence of toxicity or adverse events associated with dexamphetamine, the variation of the “fatigue” component and the “affectivity” component of the Norris Scale between inclusion and 3 months, and the variation of QOL, anxiety and depression and cognitive function between inclusion and 3 months.

Fatigue was measured using the MFI-20 as the main primary criterion,^6^ and the “fatigue” component of the Norris Visual Analog Scale (Norris scale) was used as a secondary criterion.

Depression was evaluated using the HADS scale.^7^ Affectivity was evaluated by the relevant part of the Norris Visual Analog Scale (Norris scale).^8^

QOL was measured by the EORTC QLQ-C30/BN 20 and included symptoms specific to brain tumors.

Cognitive function was measured by a standardized battery of validated tests of key domains of cognition. Tests included the Mattis Dementia Rating Scale,^9^ the Verbal Fluency-Category (VF-C) test (Animals), the Trail Making Test Parts A and B (TMT-A and TMT-B), the Wisconsin Card Sorting Test, and the Marin Apathy Scale. The VF-C test measures the speed of mental processing, verbal fluency, and executive functions. The Wisconsin Card Sorting Test evaluates verbal learning and episodic memory. The TMT-A and TMT-B measure attention, concentration, and visual-motor speed.^10^ The Wisconsin Card Sorting Test (WCST), originally developed to assess abstract reasoning ability and the ability to shift cognitive strategies in response to changing environmental contingencies, is also considered a measure of executive functions.^11^ The Grober and Buschke test evaluates episodic verbal memory.

### Treatment Compliance

Compliance was checked via a self-report notebook given to the patient, in which each patient could note the quantity of pills taken, the positive clinical effects on fatigue and the clinical side effects they observed at home between each hospital visit. It was a blank sheet to avoid the potential bias of prepopulated side-effect lists or standardized activity lists. Compliance was achieved if at least 90% of the dispensed pills were taken.

### Statistical Analysis

We expected a decrease of 25% and 5% in the MFI 20 score in the dexamphetamine group and in the placebo group, respectively. We expected that the standard deviation of this variation would not exceed 20%. We calculated that with a sample size of 18 evaluable patients in each group, the study would have a power of 80% at a two-sided significance level of 5%. The sample size was increased by 10% (20 subjects in each group) to account for the nonparametric Mann-Whitney test used for the primary analysis. Assuming a 30% rate of progressive patients, the enrollment of a maximum of 58 patients (29 subjects in each group) was planned.

The primary analysis was planned for the group of randomized patients with the nonprogressive disease within the study period, who received at least one dose of treatment (dexamphetamine or placebo), with no exclusion criteria, and with both baseline and 3-month evaluations available. Toxicities and side effects were described among subjects who received at least one dose of treatment (dexamphetamine or placebo).

Baseline characteristics in each study group were analyzed as frequencies and percentages for categorical variables and as the means and standard deviations, medians, interquartile ranges, minimum and maximum for continuous variables. Categorical variables were compared with the use of the chi-squared test or Fisher’s exact test, and continuous variables were compared with the use of Student’s *t*-test or the Mann–Whitney test, as appropriate. Longitudinal outcomes measured each month were compared between the two groups using a linear mixed-effects model (including the randomization group, time, and the interaction between group and time as fixed effects, and a random intercept). All analyses were performed at a two-sided alpha level of 5%, with R software, version 3.5.0 (R Foundation for Statistical Computing).

## Results

### Patients

**Figure 1.**
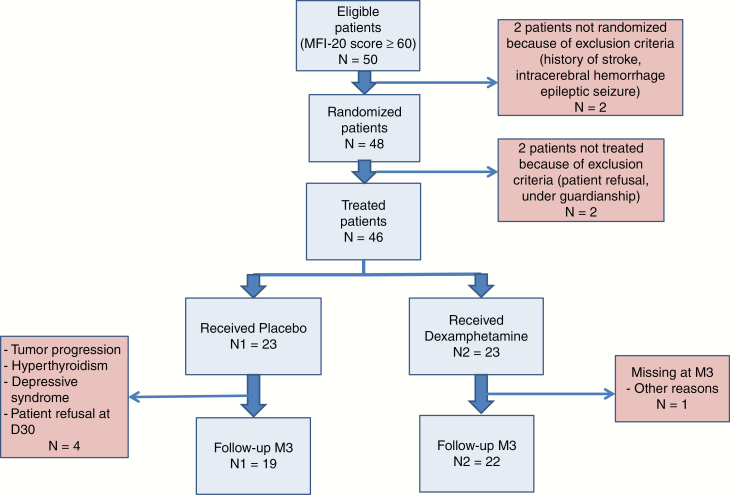
Flow chart.

From April 2013 to November 2016, a total of 46 patients from 7 different French centers were enrolled and randomized to either the dexamphetamine (DXA) group (*n* = 23) or the placebo group (*n* = 23). Because of the lower rate of tumor progression observed compared to what was initially expected (30%), it was decided to stop the study after the inclusion of 46 patients because the number of subjects required for analysis was reached. Among these 46 patients, 89% of them (41 patients) were retained for the efficacy analysis. The rate of evaluable patients was 95% (22/23) in the dexamphetamine arm and 82% (19/23) in the placebo arm at the M3 evaluation. Of those participants who dropped out of the study, the reasons for dropping out included progression of disease (*n* = 1 in the placebo arm), refusal of further therapy (*n* = 1 in the placebo arm), exclusion criteria discovered only after inclusion because they had previously been masked by the patients themselves (depression and hyperthyroid disease: 2 patients in the placebo arm), and loss to follow-up (1 participant in the DXA arm) (Figure 1).

The baseline characteristics of the 41 remaining patients are summarized by treatment arm in Table 1. The participants' ages ranged from 21 to 71 years, with a median of 57 years in the DXA arm and 51 years in the placebo arm. A majority of patients (68%, 28/41) had a KPS score ≥80%. The most common brain tumor types were gliomas of various grades, which accounted for 89% (17/19) of the patients in the placebo arm and 77% (17/22) of the patients in the DXA arm. Other diagnoses were CNS lymphomas (4 cases: 3 DXA arm and 1 placebo arm) and medulloblastomas (3 cases: 2 DXA arm and 1 placebo arm). They were previously treated by radiotherapy and chemotherapy in 34 cases (17 DXA arm, 17 placebo arm), radiotherapy alone in 3 cases (3 DXA arm, 0 placebo arm), chemotherapy alone in 4 cases (2 DXA arm, 2 placebo arm). This initial treatment was completed at a median time of 3.92 years (0.96–7.58 years) before study entry (Table 1).

**Table 1.** Baseline characteristics of the 41 evaluable patients

	Intervention arm *N* = 22	Control arm *N* = 19
Sex, *N* (%)		
Male	13 (59)	8 (42)
Female	9 (41)	11 (58)
Age (years)		
Mean (SD)	55	49
Median (IQR)	57 [45;66]	51 [41;54]
Min–max	23–71	21–69
KPS, *N* (%)		
100	3 (14)	0 (0)
90	7 (32)	11 (58)
80	4 (18)	3 (16)
70	7 (32)	4 (21)
60	1 (5)	1 (5)
Tumor status (RANO), *N* (%)		
Complete response	10 (45)	7 (37)
Partial response	0 (0)	1 (5)
Stable disease	12 (55)	11 (58)
Progressive disease	0 (0)	0 (0)
Tumor type, *N* (%)		
CNS lymphoma	3 (14)	1 (5)
Medulloblastoma	2 (9)	1 (5)
Grade II glioma	2 (9)	1 (5)
Grade III glioma	6 (27)	8 (42)
Grade IV glioma	9 (41)	8 (42)
Time from diagnosis to randomization, *N* (%)		
<1 year	2 (9)	1 (5)
≥1 year and <3 years	8 (36)	5 (26)
≥3 years and <5 years	3 (14)	3 (16)
≥5 years	9 (41)	10 (53)
Time from last treatment to randomization (years)		
Med [IQR]	3.92 [0.96–7.58]	3.78 [1.04–8.41]
Moy (std)	5.21 (5.32)	6.48 (9.23)
*N* (NA)	19 (3)	19 (0)
Proportion of patients with HADS-A >7, *N* (%)	12 (54)	5 (26)
Proportion of patients with HADS-D >7, *N* (%)	6 (27)	4 (21)
Proportion of patients with HADS-A >7 and HADS-D >7, *N* (%)	2 (9)	2 (10)
Proportion of patients with HADS-A >7 or HADS-D >7, *N* (%)	14 (63)	5 (26)

HADS-A: anxiety part of HAD scale; HADS-D: depression part of HAD scale; IQR: interquartile range.

### Treatment Compliance

The rate of compliance, defined as the use of at least 90% of the dispensed pills, was high in both study groups (77.27% in the DXA arm and 68.42% in the placebo arm, *P* = .5235).

Among the 46 randomized patients, dose information was available for 44 patients (including 22 in the DXA arm and 22 in the placebo arm), and 2 patients were released from the study due to depressive syndrome (1 placebo arm) and ischemic stroke (1 DXA arm). The number of patients who reached the full dose was 86% (38/44) divided as follows: 77% (17/22) in the DXA group and 95% (21/22) in the placebo group. The proportion of patients who tolerated the full dose was 76% (13/17) in the DXA group and 90% (19/21) in the placebo group, *P* = .38.

There were no significant differences between groups concerning concomitant medications that may affect fatigue, such as chemotherapy, steroids, anticonvulsants, or other medications.

### Toxicity

Among all the adverse events noted in the 46 randomized patients, 59% (197/333) were reported in the DXA arm and 41% (136/333) in the placebo arm. Among the 46 randomized patients, 41 patients (89%) experienced at least one side effect related to treatment. The rate of patients presenting with at least 1 adverse event was slightly different between the two groups: 100% (23/23) of patients in the DXA arm vs 78% (18/23) of patients in the placebo arm (*P* = .049) were affected. The toxicity grade was missing for only one adverse event in the DXA arm. Adverse events were mostly grade 1 toxicities (159/196 = 81%) in the DXA arm, as well as in the placebo arm (112/136 = 82%). There were a few cases of grade 2 toxicities (30/196 = 15% in the DXA arm and 22/136 = 16% in the placebo arm); grade 3 toxicities were found in 7/196 (4%) of patients of the DXA arm and 2/136 (1%) of patients of the placebo arm. A detailed analysis of the different subtypes of adverse effects described showed that the patients in the DXA arm complained more frequently about psychological side effects (mostly hyperactivity, anxiety, sleep disorder, and irritability) than the patients in the placebo arm (*P* = .018). The other parameters showed no statistically significant differences (Tables 2, 3, and 4).

**Table 2.** Descriptions of the different types of side effects among the 46 randomized patients

Categories	IA *N* = 23	PA *N* = 23	*P*
Central nervous system	16 (70%)	14 (61%)	.536
Psychiatric	**15 (65%)**	**7 (30%)**	***.018***
Cardiovascular	5 (22%)	2 (9%)	.414
Gastrointestinal	10 (43%)	7 (30%)	.359
General status	10 (43%)	10 (43%)	1.000
Ocular	3 (13%)	3 (13%)	1
Hematological	1 (4%)	2 (9%)	1
Infections	2 (9%)	2 (9%)	1
Others	0 (0%)	2 (9%)	.489

IA, interventional arm; PA, placebo arm.

**Table 3.** Repartition of all the reported side effects as a function of grade (1–2 vs 3) between the 2 arms

Grade	IA	PA	Total	*P*
1 and 2	189 (96%)	134 (99%)	323 (97%)	.318
3	7 (4%)	2 (1%)	9 (3%)	
Total	196 (59%)	136 (41%)	332 (100%)	

IA, interventional arm; PA, placebo arm.

**Table 4.** The number of patients presenting with grade 3 toxicity in each arm

	Arm of randomization			
	Dexamphetamine *N* = 23	Placebo *N* = 23	Total	*P*
Number of patients with grade 3 toxicity	3 (13%)	2 (9%)	5 (11%)	1.000

### Fatigue

A slight improvement in fatigue levels on the MFI-20 scale was observed 3 months after the beginning of treatment in both arms (median MFI-20: 70.5 at inclusion and 61 at 3 months in the DXA arm vs 70 at inclusion and 65 at 3 months in the placebo arm). There was no statistically significant difference in the variation of MFI-20 between the DXA arm and placebo arm (median variation: 14 vs 10, respectively, *P*-value: .17 (Table 5).

**Table 5.** Summary of the median (IQR 25–75) changes in primary and secondary criteria scores between inclusion and the 3-month follow-up

Scale	Arm	Baseline score	Score at M3	Score variation	*P*
MFI20 scale	Dexamphetamine	70.5 [65–80.25]	61 [50.75–67.75]	14 [4.5–24.25]	.17
	Placebo	70 [68–78.5]	65 [57–68.5]	10 [4–13]	
Trail making test A	Dexamphetamine	50 [40.5–78]	55 [39.5–73.5]	−1 [−4 to 10]	.29
	Placebo	51 [41.25–64.25]	41.5 [33.5–57.75]	6.5 [−0.75 to 17.5]	
Trail making test B	Dexamphetamine	116 [72–130]	96 [78–176]	16 [−37 to 29]]	.61
	Placebo	101.5 [82.75–117.5]	108.5 [65.75–135.75]	−2 [−16 to 21.75]	
EVA (affectivity)	Dexamphetamine	33.19 [21.91–41.31]	30.06 [21.91–43.62]	2.38 [−10.78 to 10]	.06
	Placebo	41.81 [31.69–48.94]	30.56 [20.12–41.62]	8.25 [2.34–18.09]	
EVA (Asthenia)	Dexamphetamine	47.06 [27.41–52.28]	36.25 [24.03–49.06]	6.56 [−10.88 to 13.97]	.97
	Placebo	50.62 [37–53.75]	39.56 [30.53–49.34]	4.25 [−0.06 to 12.16]	
Mattis scale	Dexamphetamine	138 [130–142]	140 [124–140]	−1 [−4 to 0]	.34
	Placebo	137 [133–141]	137 [133–142]	−2 [−7 to 1]	
Verbal fluency (semantic)	Dexamphetamine	23 [16–32]	25 [15–33]	0 [−5 to 3]	.41
	Placebo	19.5 [17–29.75]	21 [16.25–26.75]	1 [−1 to 3]	
Verbal fluency (lexical)	Dexamphetamine	17 [14–28]	19 [16–25]	−1 [−4 to 4]	.64
	Placebo	18 [12.5–25.75]	16.5 [11.5–23.75]	1 [−0.75 to 2.75]	
Marin scale (Apathy)	Dexamphetamine	57 [52–64]	54 [46–60]	2 [−2 to 11]	.46
	Placebo	58.5 [51.25–65.25]	57 [49.75–62.75]	0.5 [−6 to 2.75]	
Grober and Buschke test	Dexamphetamine	139 [130–143.25]	138.5 [124.25–142.5]	0 [−4.5 to 4]	.76
	Placebo	133 [121.75–138.5]	137.5 [123.75–142]	−0.5 [−4.25 to 2.5]	

IQR, interquartile range.

Additionally, there was no significant change over time between inclusion and M3 in the fatigue component of the Norris Scale (NS).

### Quality of Life

**Figure 2.**
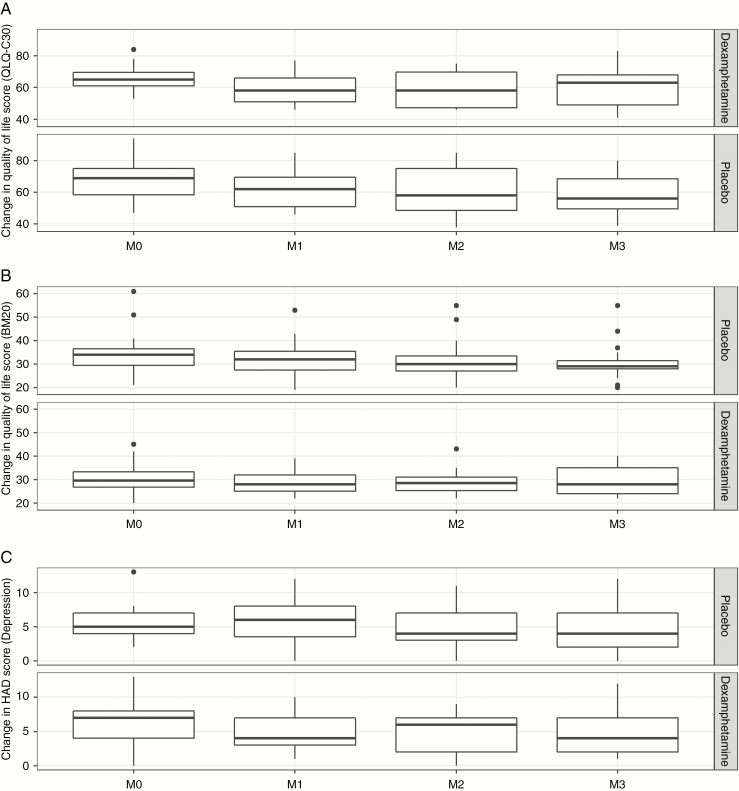
Change in QOL scores (EORTC QLQ C30: (A); BM20 module of the EORTC: (B)) and change in HADS scores (depression)—(C) between inclusion and 3-month follow-up. No significant difference was found between the two groups (test for interaction between group and time: *P*-value = .1; .06 and .77, respectively).

No statistically significant differences were found between the two arms regarding QOL outcomes (Figure 2A and B).

### Neurocognitive Functioning

No significant difference was found between the two arms regarding changes in any of the neurocognitive parameters evaluated by the Grober and Buschke test, the Trail Making Test Parts A and B, or the Marin Apathy Scale; regarding the modified Wisconsin Card Sorting Test, the data could not be analyzed because a substantial number of data points were missing (~50% of them) (Table 5).

### Depression and Anxiety

No statistically significant differences were found between the two arms regarding depression or anxiety as measured by the HADS (Figure 2C). In particular, the HADS did not capture the mild psychological changes often noted in the reports of side effects by the dexamphetamine  group.

## Discussion

Fatigue is a major issue in PBTs, affecting up to 90% of patients during the course of the disease. It has been identified as the most common symptom leading to a worsening in the QOL of brain tumor patients.^12–14^ Fatigue is believed to be of multifactorial origin, occurring as a consequence of the disease (size, location) as well as its treatment (surgery, radiotherapy, chemotherapy, steroids, antiepileptics, etc.). Several agents, including modafinil, armodafinil, and donepezil, have been tested in the hope of improving fatigue in PBT patients, but they did not achieve any significant effect.^14–19^ Among amphetamine derivatives, methylphenidate, a psychostimulant commonly used in the treatment of attention deficit/hyperactivity disorder, did not demonstrate efficacy in phase III studies of PBT patients,^20,21^ although it may be of help in noncancerous fatigue or in systemic cancers.^22,23^ Dexamphetamine was hypothesized to be a more potent stimulant than methylphenidate^24^ and has also proven useful in a controlled randomized trial of fatigue in HIV patients^5^ and in a randomized study in noncancerous chronic fatigue syndromes.^4^ On the other hand, dexamphetamine did not appear to be of help in a very brief trial (1 week) in terminally ill patients with systemic cancer.^25^

The current trial represents the first randomized trial evaluating dexamphetamine in PBT patients suffering from fatigue. To focus the trial on “true” fatigue, we excluded patients with severe depression (HADS score < 8) to avoid biases concerning this issue.

Because major methodological concerns were raised in reviews evaluating interventions to improve fatigue in this population, we took care to select a design (placebo-controlled randomized trial) in line with the recommendations of experts.^14,26^ Moreover, dexamphetamine was used at a relatively high dose (30 mg) for a substantial period (3 months) in patients with acceptable performance status (KPS > 60) and a responsive or stable PBT.

Unfortunately, we could not show an effect of dexamphetamine on fatigue in comparison with a placebo. Dexamphetamine also had no significant effect on other scales or batteries evaluating depression, anxiety, cognitive functioning, and QOL.

Thus, to date, as noted by a recent Cochrane review, pharmacological interventions to relieve fatigue in PBT remain unsuccessful.^2^

Drug tolerance was better than expected. At the onset of the trial, we (clinicians, patients, and relatives) feared the potential risks of neurologic, psychiatric, and cardiac side effects of dexamphetamine in this fragile population.^27^ In the experiment, however, we reported no grade 4 toxicity, no related deaths, no severe impairment of epileptic seizure balance and no dependence on the drug at the end of the study. The most remarkable severe side effect observed in the dexamphetamine group was a posterior cerebral artery stroke (grade 3 toxicity) in one patient. This patient also had marked radiation-induced leukopathy and suspected radiation-induced vasculopathy. However, it should be noted that more patients in the dexamphetamine group than in the placebo group reported psychological side effects, but they were mildly tolerable and reversible impairments.

Recruitment of patients was more difficult than expected. Half of the eligible patients declined to participate. Here, once again, the fear of side effects also played a role; reading the informed consent form that detailed all the possible effects of amphetamines, including the very rare occurrence of “sudden death,” discouraged many of them. Other limiting factors were the frequent required visits to the hospital (3 times the first month, then every month) as well as the driving prohibition and consequent difficulties tor travel due to the legal status of the drug in France. The difficulty of recruiting patients for “quality-of-life” trials evaluating pharmacologic agents is now well recognized.^19^

There are several limitations in our study, such as the heterogeneous population of brain tumors, including gliomas, medulloblastomas, and CNS lymphomas, which had been treated with different treatment schemes, which may have influenced the response to an intervention for fatigue; however, most of them (83%, 34/41) were previously treated by radiotherapy and chemotherapy. The choice of MFI 20 may be another limitation. Many tools have been developed to measure fatigue in patients suffering from cancer; we decided to choose the MFI score because of the longstanding use of it in our department to evaluate patients in daily practice as well as the clinical research with this specific tool. Finally, our choice of a cut-off of 60 on the global MFI 20 scale may also be criticized. However, we deliberately selected a population of patients complaining of severe and objective fatigue for our target population in which a potentially dangerous agent was to be tested. We are convinced (and this is also a key issue described in the literature^25^) that it is necessary to have a high fatigue level as a mandatory inclusion criterion for studies on this topic. Because a clear definition of a high degree of fatigue is often lacking in the literature, we conducted a small study in our department before beginning this trial, including patients and healthy subjects (*personal data, unpublished*), to define an objective global score cut-off on the MFI 20 and that is the reason why we decided to choose a cut-off of 60. Finally, the length of the observation period may also be considered as potential limitation although we took care to have a relatively long period of observation in contrast with previous studies.^4,5,25^

The disappointing results of pharmacological interventions to date should not discourage continuous and tenacious research in this area, which remains a key issue in PBT patients.

For example, a recent meta-analysis suggested that exercise and psychological interventions are effective for reducing cancer-related fatigue during and after cancer treatment and seemed to be significantly better than the available pharmaceutical options.^28^ Whether these strategies may also help to reduce fatigue in PBT patients remains to be evaluated.

## Conclusion

With the dosing schedule used in this placebo-controlled randomized study in PBT patients, dexamphetamine did not improve fatigue, anxiety, depression, cognitive function, or QOL.

## Funding

AP-HP was the promotor of this study **(ClinicalTrials.gov Identifier: NCT02363075)**. This study was supported by the ARTC (“Association pour la Recherche sur les Tumeurs Cérébrales”), which funded the study and provided the experimental drug and placebo.

## 

*Conflict of interest statements*. All authors have no conflict of interest.

## Author Contributions

Florence Laigle-Donadey, Mamadou Hassimiou Diallo, David Hajage and Jean-Yves Delattre wrote the manuscript. François Ducray, Matthieu Boone, Carole Ramirez, Olivier Chinot, Damien Ricard reviewed the consecutive drafts and provided input. All authors edited and approved the final version of the manuscript.
